# Pharmacological stimulation of NQO1 decreases NADPH levels and ameliorates acute pancreatitis in mice

**DOI:** 10.1038/s41419-018-1252-z

**Published:** 2018-12-18

**Authors:** AiHua Shen, Hyung-Jin Kim, Gi-Su Oh, Su-Bin Lee, SeungHoon Lee, Arpana Pandit, Dipendra Khadka, Subham Sharma, Seon Young Kim, Seong-Kyu Choe, Sei-Hoon Yang, Eun-Young Cho, Hyuk Shim, Raekil Park, Tae Hwan Kwak, Hong-Seob So

**Affiliations:** 1 Center for Metabolic Function Regulation & Department of Microbiology, Wonkwang University School of Medicine, Iksan, Jeonbuk 54538 Republic of Korea; 2 Internal Medicine, Wonkwang University School of Medicine, Iksan, Jeonbuk 54538 Republic of Korea; 3 Department of Biomedical Science & Engineering, Institute of Integrated Technology, Gwangju Institute of Science and Technology, Gwangju, 61005 Republic of Korea

## Abstract

Reactive oxygen species (ROS) regulates the activation of inflammatory cascades and tissue damage in acute pancreatitis. NADPH oxidase (NOX) is upregulated in pancreatitis and is one of the major enzymes involved in ROS production using NADPH as a general rate-limiting substrate. Dunnione, a well-known substrate of NAD(P)H:quinone oxidoreductase 1 (NQO1), reduces the ratio of cellular NADPH/NADP^+^ through the enzymatic action of NQO1. This study assessed whether a reduction in cellular NADPH/NADP^+^ ratio can be used to regulate caerulein-induced pancreatic damage associated with NOX-induced ROS production in animal models. Dunnione treatment significantly reduced the cellular NADPH/NADP^+^ ratio and NOX activity through the enzymatic action of NQO1 in the pancreas of the caerulein-injection group. Similar to these results, total ROS production and expressions of mRNA and protein for NOX subunits Nox1, p27^phox^, p47^phox^, and p67^phox^ also decreased in the dunnione-treated group. In addition, caerulein-induced pancreatic inflammation and acinar cell injury were significantly reduced by dunnione treatment. This study is the first to demonstrate that modulation of the cellular NADPH:NADP^+^ ratio by enzymatic action of NQO1 protects acute pancreatitis through the regulation of NOX activity. Furthermore, these results suggest that modulation of the NADPH:NADP^+^ ratio in cells by NQO1 may be a novel therapeutic strategy for acute pancreatitis.

## Introduction

Acute pancreatitis is a complicated disease characterized by the activation of digestive enzymes, infiltration of neutrophils, and necrosis of the pancreatic acinar cells^1^. Based on high serum levels of cholecystokinin (CCK) observed in patients with acute pancreatitis, injecting high doses of caerulein (CCK8 analogue) into mice causes many symptoms similarly observed in human pancreatitis such as excessive pancreatic secretion of amylase and lipase, cytoplasmic vacuolization, necrosis of acinar cells, edema, and pancreatic infiltration of neutrophils^2^.

Reactive oxygen species (ROS) are involved in the pathogenesis and development of acute pancreatitis. In addition, previous studies have reported that NADPH oxidase (NOX) is a major source of ROS in acute pancreatitis^3,4^. NOX is the transmembrane flavoprotein enzyme that catalyzes the univalent reduction of oxygen by using NADPH as an electron donor to make the superoxide free radical. NOX is a multimeric enzyme consisting of five different subunits, including NOX (*e.g*., NOX1), NOX organizer (NOXO; *e.g*., p47phox), and NOX activator (NOXA; *e.g*., p67phox), p22phox, and p40 phox. Among them, NOX1, p22phox, p47phox, and p67phox are expressed in pancreatic acinar cells and are closely related to caerulein-induced acute pancreatitis^5^.

NAD(P)H:quinone oxidoreductase 1 (NQO1) is a cytosolic antioxidant flavoprotein that catalyzes the oxidation of NAD(P)H to NAD(P)^+^ by various quinones^6^. Dunnione, an orange-red pigment obtained from *Streptocarpus dunnii* Mast, was initially identified as an anti-fungal agent^7,8^, but has recently been shown to act as a substrate of NQO1, thereby decreasing the NAD(P)H/NAD(P)^+^ ratio through NQO1-mediated NAD(P)H oxidation^9–11^. Interestingly, a previous study have shown that decreasing the NAD(P)H/NAD(P)^+^ ratio by NQO1 suppressed cisplatin-induced ototoxicity and salt-induced renal injury via the modulation of NOX-derived ROS generation in an animal model^10,12^. In this study, we investigated the effects of dunnione on caerulein-induced pancreatic injury and demonstrated that decreased levels of cellular NADPH resulting from the enzymatic action of NQO1 using dunnione as a substrate ameliorated pancreatic damage through the modulation of NOX enzyme activity.

## Materials and methods

### Reagents

Dunnione was chemically synthesized by Erum Biotechnologies (Suwon, Korea) and micronized as particles to enhance oral bioavailability. Caerulein was purchased from Sigma Chemical Co (Sigma, St Louis, MO, USA). ML171 was purchased from R&D Systems (Minneapolis, MN, USA). Antibodies against NOX1, p22phox, p47phox, p67phox, and β-actin were purchased from Santa Cruz Biotech Inc. (Santa Cruz, CA, USA). DMEM, FBS, and other tissue culture reagents were obtained from Life Technologies Inc. (Gaithersburg, MD, USA).

### Animals

Male C57BL/6 mice were obtained from the Central Laboratory Animal Inc. (Seoul, Korea). NQO1 knockout (NQO1^−/−^) mice on a C57BL/6 background were kindly provided by Dr. C. H. Lee (Animal Model Center, Korea Research Institute of Bioscience and Biotechnology, Daejeon, Korea). The NQO1^−/−^ mice did not show any developmental abnormalities. All mice were fed a standard commercial diet while housed at an ambient temperature of 20–22 °C with a relative humidity of 50 ± 5% under a 12/12 h light–dark cycle in a specific pathogen-free facility. Experiments were performed in 8-week-old mice weighing between 20 and 25 g, and all mice were age matched to within 3 days. All of the animal studies were approved by the Animal Care and Use Committee at Wonkwang University School of Medicine.

### Experimental design for acute pancreatitis

C57BL/6 and NQO1^−/−^ mice were fasted for 17 h before the treatment but provided with free access to water. Acute pancreatitis was induced by six injections of caerulein (50 μg/kg, intraperitoneal [i.p.] at intervals of 1 h) as described previously. Each experimental group was composed of five mice. The control group received an i.p. injection of saline (0.9% NaCl) solution. In the caerulein and dunnione combined groups, three doses of dunnione (10, 20, and 40 mg of dunnione/kg bodyweight) dissolved in vehicle (corn oil) were orally injected at 3 h before the first caerulein injection. All mice were sacrificed at 6 h after the last caerulein injection. Blood samples were taken to determine the serum amylase, lipase, and cytokine levels. A portion of the pancreas was fixed overnight in 4% paraformaldehyde in phosphate-buffered saline (PBS, pH 7.4) at 4 °C for immunohistochemical studies, embedded in paraffin, cut into 4-μm thick sections, which were then stained with hematoxylin and eosin (H&E) to observe the morphological changes under a light microscope by standard procedures. After staining with H&E, histological injury score of pancreatic slides were graded in a blinded manner without knowledge of the experimental design according to the severity and extent of edema, inflammatory cell infiltration, and acinar necrosis as described in Table 1. A portion of the pancreas was also frozen in liquid nitrogen for western blotting and RT-PCR.

### Serum α-amylase and lipase assay

Levels of α-amylase and lipase in the serum were measured with a QuantiChrom Lipase assay kit (DLPS-100; BioAssay Systems, CA, USA) and a QuantiChrom α-Amylase assay kit (DAMY-100; BioAssay Systems, CA, USA) according to the manufacturer’s instructions.

### Acinar cell isolation

Pancreatic acini were isolated from C57BL/6 mice using collagenase digestion. Pancreatic tissue was minced with scissors and digested for 15 min in PSA solution (140 mmol NaCl, 10 mmol HEPES, 5 mmol KCl, 1 mmol MgCl_2_, 1.5 mmol CaCl_2_, 10 mmol sodium pyruvate, 10 mmol ascorbate, 10 mmol glucose, 0.1% bovine serum albumin, 0.01% soybean trypsinogen inhibitor, and 150 units of collagenase/ml). Cells were continuously shaken and gassed with 100% O_2_ in a 37 °C water bath and subsequently washed in fresh isolation medium. After collagenase digestion, the tissue was gently pipetted. Dispersed acini were filtered through a 150-μm nylon mesh, centrifuged 3 times (each for 60 s at 1000 r.p.m.), resuspended in Waymouth medium (Invitrogen) and incubated with 95% O_2_ and 5% CO_2_ for 4 h.

### Western blot analysis

Total protein from pancreatic tissue was extracted in ice-cold lysis buffer, and the contents were measured using the Bio-Rad protein assay kit (Bio-Rad Laboratories, Hercules, CA, USA). Twenty micrograms of protein were then subjected to electrophoresis on 10% SDS-polyacrylamide gels for 3 h at 20 mA, after which the protein was transferred to a nitrocellulose membrane. The membrane was then incubated in 5% (wt/vol) dried milk protein in PBS containing 0.05% (vol/vol) Tween-20 (PBS-T) for 1 h, washed in PBS-T, and then further reacted with the primary antibody (1:1,000) for 1 h. Next, the membrane was extensively washed with PBS-T and incubated with the appropriate secondary antibodies for 1 h at room temperature. After extensive washes, protein bands on the membrane were visualized using chemiluminescent reagents according to the manufacturer’s instructions (Supersignal Substrate; Pierce, Rockford, IL).

### Measurement of IL-1β proinflammatory cytokine

To measure the serum level of the IL-1β proinflammatory cytokine, whole blood was isolated from mice before sacrifice, incubated at 4 °C for 16 h, and centrifuged at 4000 rpm for 20 min. Thereafter, the level of IL-1β was determined by ELISA (ELISA, Quantikine Kit; R&D Systems, Minneacute pancreatitisolis, MN) according to the manufacturer’s instructions.

### Quantitative Real-time PCR (qRT-PCR) analysis

Total RNA was isolated from cells using TRIzol (Invitrogen, CA, USA) according to the manufacturer’s protocol. Three micrograms of RNA were converted into cDNA using the First Strand cDNA Synthesis Superscript kit (Invitrogen) according to the manufacturer’s protocol. Quantitative real-time PCR was performed using SYBR Green (Invitrogen). Reactions were performed in triplicate and the specificity was monitored using melting curve analysis after cycling. The primers used were as follows: IL-1β, 5′-TCTTTGAAGTTGACGGACCC-3′ and 5′-TGAGTGATACTGCCTGCCTG-3′; MCP1, 5′-GCTGGAGAGCTACAAGAGGATCA-3′ and 5′-ACAGACCTCTCTCTTGAGCTTGGT-3′; p22phox, 5′- GTGGACTCCCATTGAGCCTA-3′ and 5′-CTCCTCTTCACCCTCACTCG-3′; p47phox, 5′- GTCCCTGCATCCTATCTGGA-3′ and 5′-ATGACCTCAATGGCTTCACC-3′; p67phox, 5′- TCTCATGCATGCCAAGAAAG-3′ and 5′-CTTCATGTTGGTTGCCAATG-3′; NOX1, 5′- CTTGCACCHATTGCTTTTTAT-3′ and 5′-CATTAGATGGGTGCATGACAA-3′; GAPDH, 5′-TCCCACTCTTCCACCTTCGA-3′ and 5′-AGTTGGGATAGGGCCTCTCTTG-3′. Relative mRNA expression was quantified using the ΔΔCt method and GAPDH was used as an internal control. The results were expressed as fold change.

### Comet assay

The comet assay was performed under alkaline conditions using the OxiSelectTM Comet assay kit (Cell Biolabs Inc. San Diego, CA, USA) following the manufacturer′s instructions. Briefly, electrophoresis was carried out on 1 × 10^5^ cells layered on comet slides for 30 min at 25 V and 300 mA. Then the slides were stained with 100 μL/well of diluted Vista Green DNA Dye (Cell Biolabs Inc.). The comet images were captured with an Olympus IX71 fluorescence microscope (200X magnification). The distance of DNA migration from each cell was measured from the body of the nuclear core to the trailing edge of the comet. The comet lengths of 50 individual cells were measured for each treatment group.

### Assessment of NOX activity

NOX activity was quantified based on the reduction of cytochrome c in pancreatic acinar cells. Reduction of cytochrome c was determined by reading the absorbance changes at 550 nm for 3 min in the presence or absence of the NOX inhibitor diphenyleneiodonium (DPI; 100 μM). DPI-inhibitable activity [(absorbance changes in the absence of DPI)−(absorbance changes in the presence of DPI)] was taken as NOX activity. The results were expressed as fold change.

### Determination of ROS production

To determine total ROS production, pancreatic tissue extracts (100 mL) from each experimental group were incubated with 20 μM H2-DCFDA (Invitrogen, San Diego, CA, USA) for 60 min at 37 °C. Fluorescence intensity was recorded using the CytoFluor series 4000 fluorometer (PerSeptive Biosystems Inc.) and normalized to protein content. For measurement of intracellular ROS in pancreatic acinar cells, the cells (5 × 10^5^ / ml) in a chamber slide (Nalge Nunc, Naperville, IL, USA) were cultured in the absence or presence of caerulein (10 nM) for 60 min, and then further incubated with 20 μM H2-DCFDA (Invitrogen, San Diego, CA, USA) for 60 min at 37 °C. Fluorescence intensity was recorded using the CytoFluor series 4000 fluorometer (PerSeptive Biosystems Inc.).

### NQO1 activity assay

NQO1 activity was analyzed by measuring the conversion rate of NADH to NAD^+^ using DCPIP (2,6-dichlorophenolindophenol; Sigma) as a substrate. The reduction of NADH was measured at 600 nm over 2 min. NQO1 activity was measured in a 1-ml reaction volume containing 200 μmol/l NADH (Sigma), 40 μmol/l DCPIP, and Tris-HCl buffer (25 mmol/l Tris-HCl, pH 7.4 and 0.7 mg/ml bovine serum albumin). Moreover, dicumarol (Sigma) was used to inhibit NQO1 activation.

### Statistical analysis

Each experiment was performed at least three times, and all values represent the means ± S.D. of triplicate analyses. One-way ANOVA was used to analyze the statistical significance of the results, and values of *p* < 0.05 were considered to be statistically significant.

## Results

### Dunnione ameliorates caerulein-induced acute pancreatitis

**Fig. 1:**
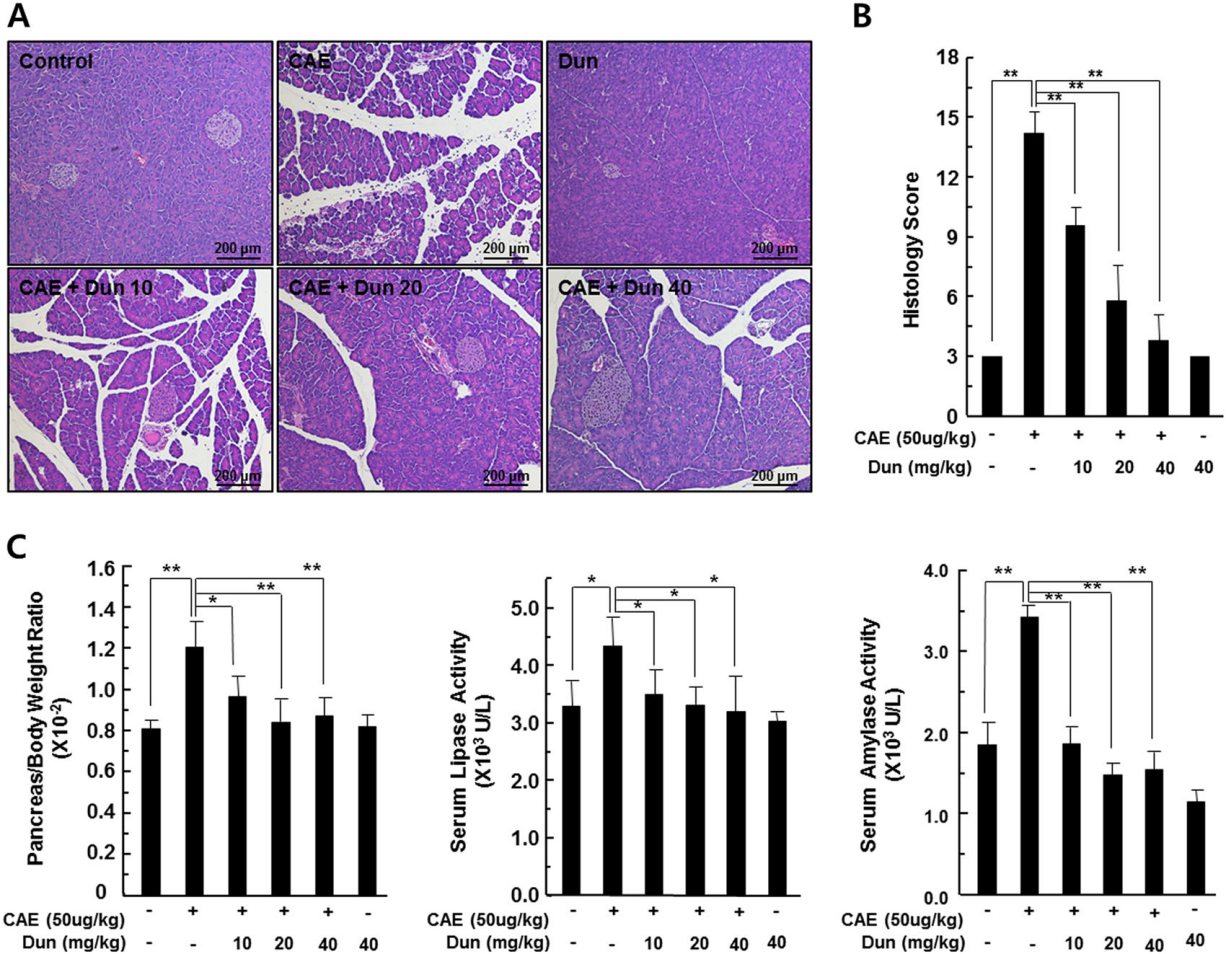
Effect of dunnione on caerulein-induced acute pancreatitis in wild-type mice. **a**, **b** Pancreas injury was estimated by hematoxylin and eosin staining and a histologic damage score. Cont, saline (0.9% NaCl)-treated control group; CAE, 50 µg/kg caerulein-only group; CAE + Dun 10, caerulein plus 10 mg/kg dunnione combined group; CAE + Dun 20, caerulein plus 20 mg/kg dunnione combined group; CAE + Dun 40, caerulein plus 40 mg/kg dunnione combined group; Dun, 40 mg/kg dunnione-only group. Scale bar: 200 μm. **c** The pancreas-to-bodyweight ratio and serum amylase/lipase levels were measured. Each value represents the mean ± SD (n = 5). *P < 0.05, **P < 0.01

C57BL/6 mice were treated with caerulein, dunnione, or dunnione plus caerulein to investigate the effect of dunnione on caerulein-induced acute pancreatitis. Histologically, caerulein administration caused moderately severe pancreatitis characterized by pancreatic interstitial edema, cellular swelling, inflammatory cell infiltrations, and parenchymal necrosis. However, each of these pathological parameters was strongly reduced by dunnione in a dose-dependent manner (Fig. 1a). In addition, semiquantitative evaluation of pancreatitis including edema, cell death, and inflammatory cell infiltration showed that the degree of pancreatitis due to caerulein was reduced by dunnione (Fig. 1b). Pancreatic injury was also assessed by comparing the pancreas-to-bodyweight ratio and serum levels of amylase/lipase in the experimental groups. Caerulein treatment significantly increased the pancreas-to-bodyweight ratio and serum levels of amylase/lipase, while dunnione attenuated the effect of caerulein in a dose-dependent manner (Fig. 1c).

### Dunnione inhibits ROS generation through the regulation of NADPH oxidase

**Fig. 2:**
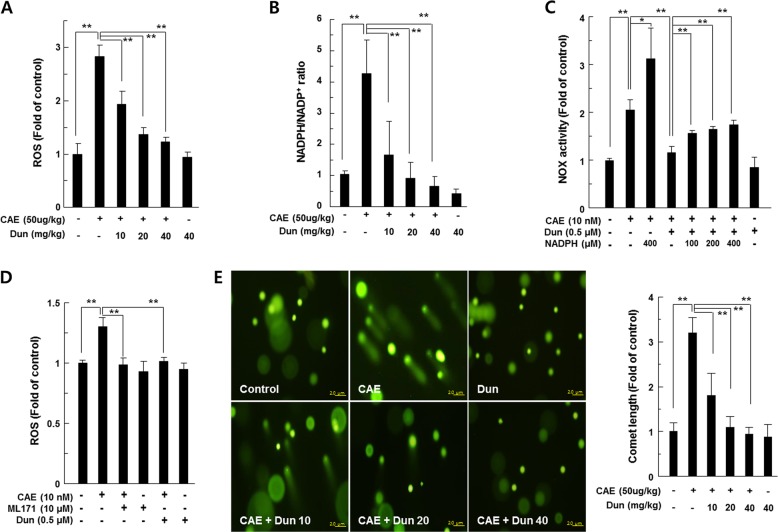
Effect of dunnione on the NADPH/NADP^+^ ratio and production of intracellular reactive oxygen species (ROS) during caerulein-induced acute pancreatitis. **a** Pancreatic tissue was isolated 11 h after the final injection of caerulein in wild-type (WT) mice. Tissue extracts were incubated with 20 μM of H2-DCFDA at 37 °C for 60 min, and then ROS levels were measured using a fluorometer. **b** NADPH and NADP^+^ were extracted from pancreatic tissues of caerulein-treated WT mice, and changes in the NADPH/NADP^+^ ratio were measured using the NADPH/NADP^+^ assay kit. **c** Freshly prepared pancreatic acinar cells were treated with NADPH or dunnione and cultured in the presence of caerulein for 12 h. NOX activity was measured by cytochrome c reduction assay. **d** Freshly prepared pancreatic acinar cells were treated with ML-171 or dunnione and cultured in the presence of caerulein for 12 h. ROS levels were measured using a fluorometer and normalized to protein content. **e** Representative images and quantification of damaged DNA in the comet assay. Cont, saline (0.9% NaCl)-treated control group; CAE, 50 µg/kg caerulein-only group; CAE + Dun 10, caerulein plus 10 mg/kg dunnione combined group; CAE + Dun 20, caerulein plus 20 mg/kg dunnione combined group; CAE + Dun 40, caerulein plus 40 mg/kg dunnione combined group; Dun, 40 mg/kg dunnione-only group. Each value represents the mean ± SD (n = 5). *P < 0.05, **P < 0.01. Scale bar: 20 μm

**Fig. 3:**
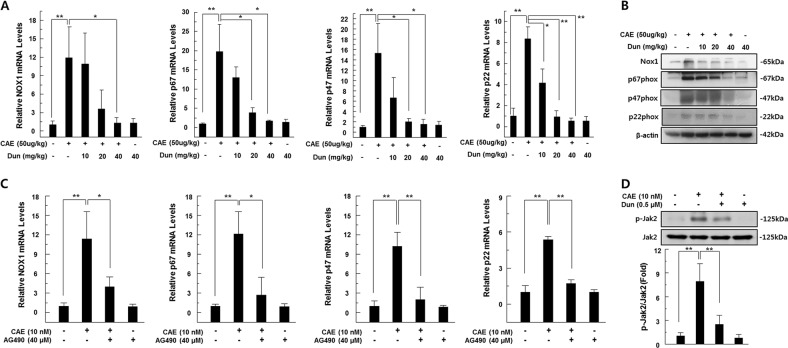
Effect of dunnione on the expression of pancreatic NADPH oxidase isoforms in caerulein-induced acute pancreatitis. **a**, **c** Pancreatic mRNA levels of NOX1, p67phox, p47phox, and p22phox measured using qRT-PCR. **b** Pancreatic protein levels of NOX1, p67phox, p47phox, and p22phox analyzed by western blotting. **d** Protein levels of p-Jak2 and Jak2 analyzed by western blotting. Each value represents the mean ± SD (*n* = 5). *P < 0.05, **P < 0.01

ROS are a major pathogenic factor of acute pancreatitis and causes DNA damage and contributes to the death of pancreatic acinar cell^13^. Thus, we measured ROS levels in the pancreatic tissues of caerulein-injected mice using the peroxide-sensitive fluorescent probe H2-DCFDA and detected DNA damage using the comet assay. We observed a significant increase in ROS levels in the pancreatic tissue of caerulein-treated mice compared to saline-injected control mice. However, dunnione treatment significantly reduced ROS production in acute pancreatitis in a dose-dependent manner (Fig. 2a). Previous studies have shown that NOX is a major source of ROS in acute pancreatitis and is regulated by NADPH, a rate-limiting substrate for NOX activation^12,14^. Meanwhile, dunnione is known as a substrate of NQO1 and also decreases the cellular NADPH/NADP^+^ ratio^10,11^. Therefore, we examined the effects of dunnione on the cellular ratio of NADPH/NADP^+^ in the experimental groups. As shown in Fig. 2b, the cellular NADPH/NADP^+^ ratio was significantly increased in the pancreatic tissues of acute pancreatitis, but this increase was significantly reduced by dunnione (Fig. 2b). In addition, exogenously added NADPH is associated with increased NOX activity (Fig. 2c). Furthermore, dunnione and the NOX1-specific inhibitor ML171 attenuated the increased ROS levels by caerulein (Fig. 2d). Dunnione also inhibited caerulein-induced DNA damage (Fig. 2e). RT-PCR and western blotting results revealed that caerulein increased mRNA and protein levels of NOX1 and NOX1-related subunits, including p22phox, p47phox and p67phox, which are involved in the activation of NOX1. However, the dunnione treatment resulted in a dose-dependent decrease in mRNA and protein expression of all factors involved in the activation of NOX1 (Fig. 3a, b). Previous studies have shown that janus tyrosine kinase 2 (Jak2) is an important regulator of NOX expression and is activated by caerulein in pancreatic acinar cells^15,16^. Thus, we investigated the effect of dunnione on Jak2 activation in caerulein-treated pancreatic acinar cells. As shown in Fig. 3d, dunnione inhibited caerulein-induced phosphorylation of Jak2 in pancreatic acinar cells. We also found that caerulein-induced NOX mRNA expression was downregulated by treatment with the Jak2 inhibitor AG490 (Fig. 3c). These observations suggest that dunnione has an inhibitory effect on NOX expression through a decrease in Jak2 activation in acute pancreatitis.

### Dunnione attenuates inflammatory signaling

**Fig. 4:**
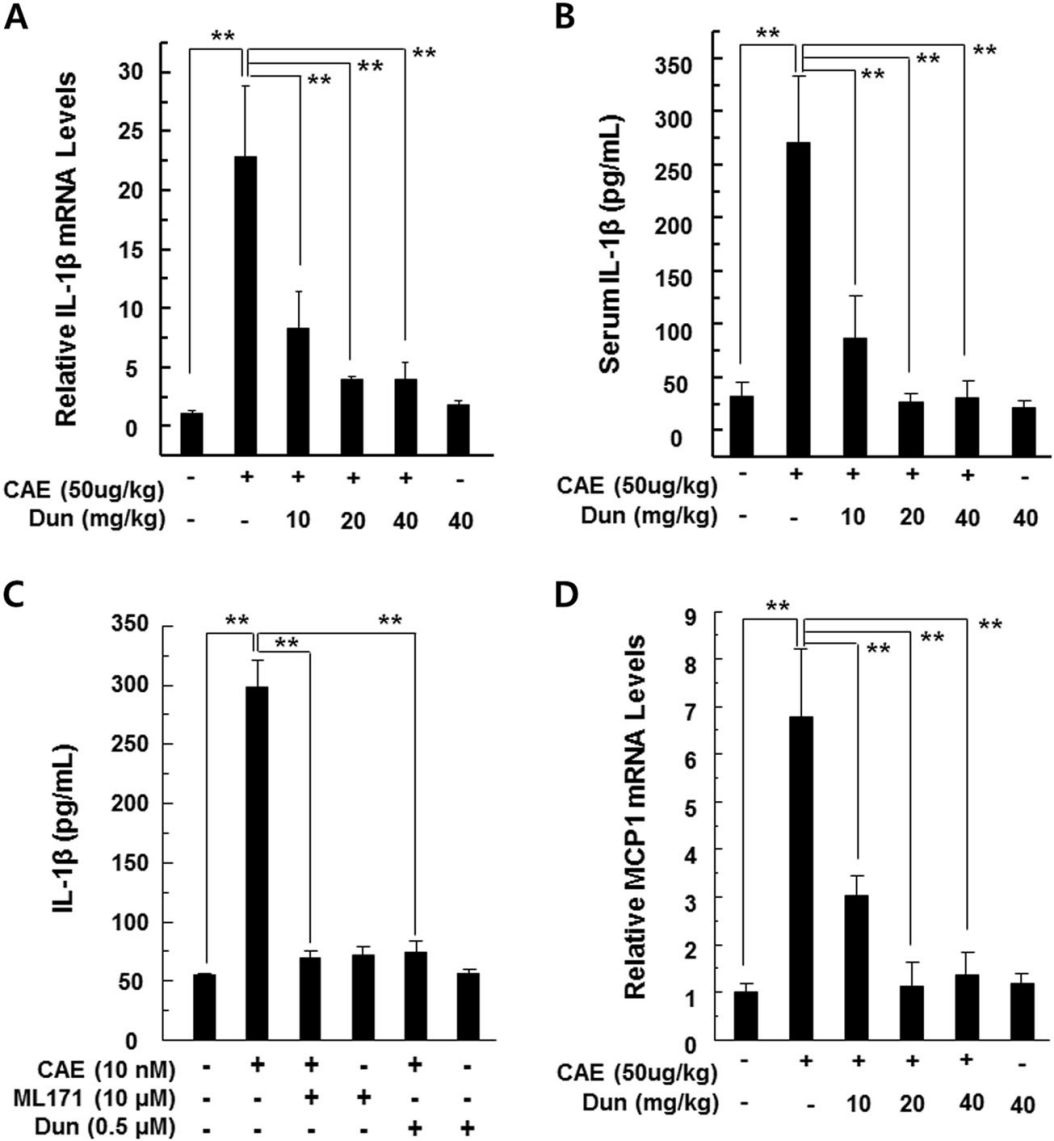
Effect of dunnione on inflammatory changes during caerulein-induced AP. **a**, **b** Levels of pancreatic IL-1β mRNA and serum IL-1β protein were measured by qRT-PCR and ELISA. **c** Freshly prepared pancreatic acinar cells were treated with ML-171 or dunnione and cultured in the presence of caerulein for 12 h. IL-1β secretion in culture supernatants determined by ELISA. **d** Levels of pancreatic MCP-1 mRNA were measured by qRT-PCR. Each value represents the mean ± SD (*n* = 5). **P < 0.01

Serum protein and tissue mRNA levels of IL-1β were assessed because it was known that IL-1β is a key effector cytokine and is required for complete pancreatic damage and inflammation in acute pancreatitis^17,18^. A substantial increase in IL-1β mRNA and protein was observed in pancreatic samples and sera collected from caerulein-treated mice compared to saline-injected control mice. In contrast, serum and pancreatic levels of IL-1β were significantly reduced in dunnione-treated mice (Fig. 4a, b). Thus, to investigate the direct involvement of NOX1 in IL-1β secretion, freshly isolated pancreatic acinar cells were pretreated with NOX1-specific inhibitor ML171 or dunnione and then stimulated with caerulein. We observed a significant increase in the extracellular levels of IL-1β in pancreatic acinar cells treated with caerulein. However, this increase was significantly reduced by ML171 or dunnione (Fig. 4c). We also evaluated MCP-1, another well-known proinflammatory chemokine involved in acute pancreatitis, in pancreatic tissue after the caerulein injection. As shown in Fig. 4d, dunnione administration significantly reduced caerulein-induced MCP-1 mRNA expression.

### The protective effect of dunnione on acute pancreatitis required NQO1

**Fig. 5:**
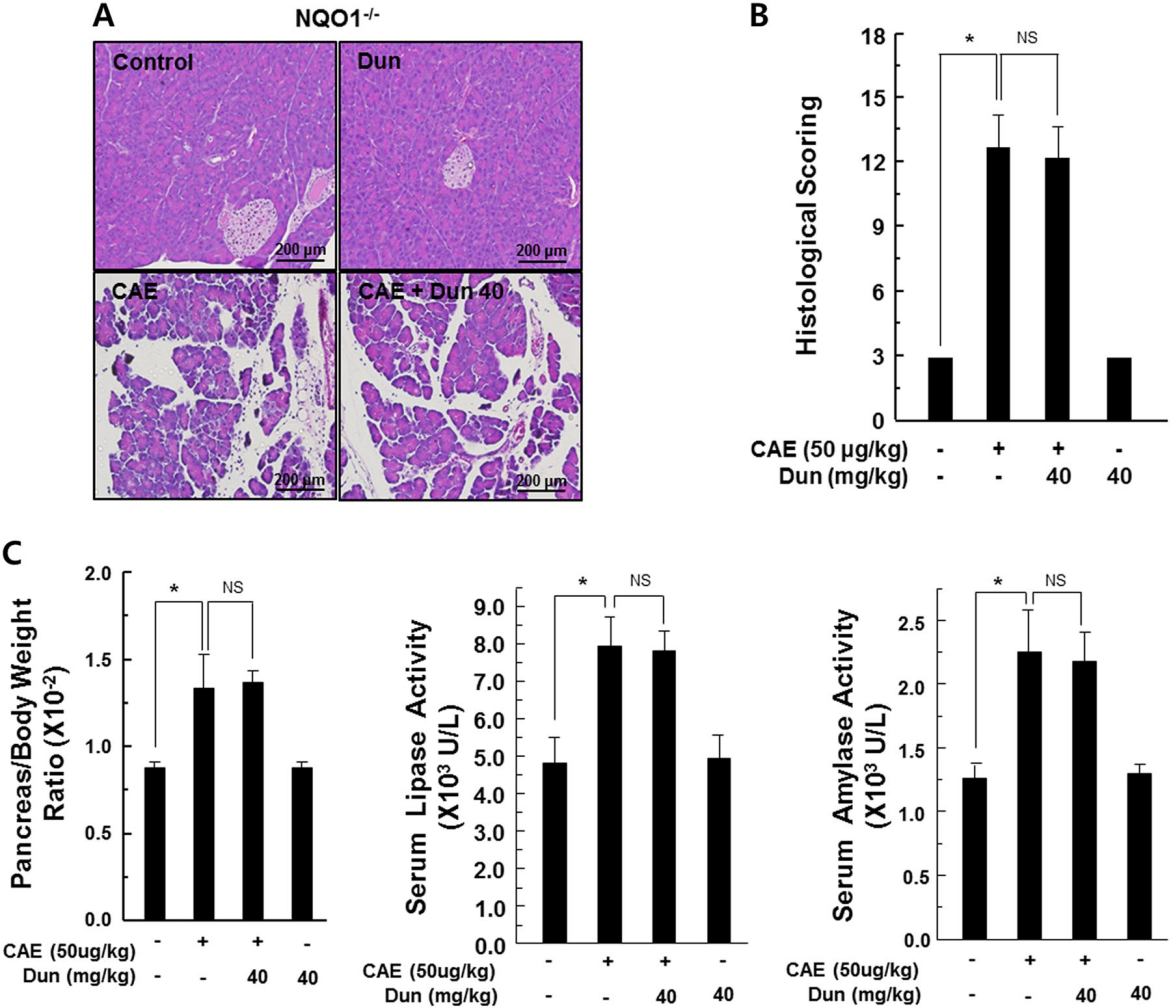
Effect of dunnione on caerulein-induced acute pancreatitis in NQO1^−/−^ mice. **a**, **b** Pancreas injury in NQO1^−/−^ mice was estimated by hematoxylin and eosin staining and histologic damage scores. Cont, saline (0.9% NaCl)-treated control group; CAE, 50 µg/kg caerulein-only group; CAE + Dun 10, caerulein plus 10 mg/kg dunnione combined group; CAE + Dun 20, caerulein plus 20 mg/kg dunnione combined group; CAE + Dun 40, caerulein plus 40 mg/kg dunnione combined group; Dun, 40 mg/kg dunnione-only group. Scale bar: 200 μm. **c** The pancreas-to-bodyweight ratio and serum amylase/lipase levels were measured. Each value represents the mean ± SD (*n* = 5). *P < 0.05, NS not significant

**Fig. 6:**
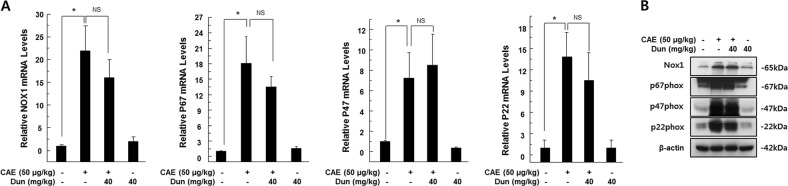
Effect of dunnione on the expression of pancreatic NADPH oxidase isoforms during caerulein-induced acute pancreatitis in NQO1^−/−^ mice. **a** Pancreatic mRNA levels of NOX1, p67phox, p47phox, and p22phox measured using qRT-PCR. **b** Pancreatic protein levels of NOX1, p67phox, p47phox, and p22phox analyzed by western blotting. Each value represents the mean ± SD (*n* = 5). *P < 0.05, NS not significant

**Fig. 7:**
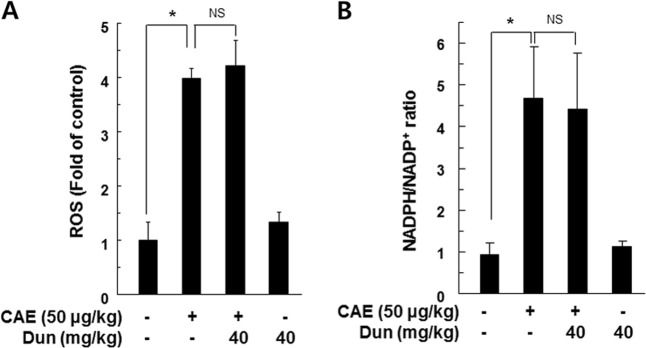
Effect of dunnione on the production of reactive oxygen species (ROS) and NADPH/NADP^+^ ratio during caerulein-induced AP in NQO1^−/−^ mice. **a** Pancreatic tissue was isolated 11 h after the final injection of caerulein in NQO1^−/−^ mice. Tissue extracts were incubated with 20 μM of H2-DCFDA at 37 °C for 60 min, and then ROS levels were measured using a fluorometer. **b** NADPH and NADP^+^ were extracted from pancreatic tissues of caerulein-treated NQO1^−/−^ mice, and changes in the NADPH/NADP^+^ ratio were measured using the NADPH/NADP^+^ assay kit. *P < 0.05. NS not significant

**Fig. 8:**
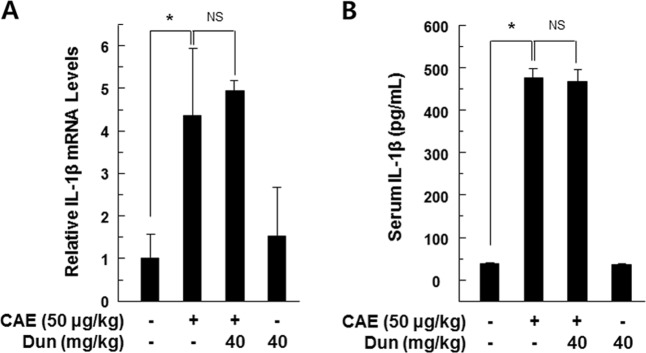
Effect of dunnione on inflammatory changes during caerulein-induced acute pancreatitis in NQO1^−/−^ mice. Levels of pancreatic IL-1β mRNA (**a**) and serum IL-1β protein (**b**) were measured by qRT-PCR and ELISA. Each value represents the mean ± SD (*n* = 5). *P < 0.05. NS not significant

We investigated whether the protective effect of dunnione was mediated by NQO1 activation. As shown in supplementary Fig. S1, NQO1 activity was significantly increased in dunnione-treated pancreatic acinar cells, whereas it was attenuated to the control level by the addition of the NQO1 inhibitor dicumarol. Interestingly, dicumarol itself completely abrogated NQO1 activity (Supplementary Fig. S1). In addition, dunnione treatment significantly reduced caerulein-induced NOX activity and cellular ROS levels in pancreatic acinar cells. However, this reduction was significantly inhibited by dicumarol (Supplementary Fig. S2A–B). Furthermore, dicumarol treatment significantly reduced the protective effects of dunnione on DNA damage and cytotoxicity by caerulein (Supplementary Fig. S2C–D). Next, we performed a series of experiments using NQO1^−/−^ mice, similar to those shown in Figs. 1–4 using wild-type (WT) mice. First, the injection of caerulein in NQO1^−/−^ mice showed a typical acute pancreatitis with histological staining. However, unlike the results from WT mice, dunnione failed to prevent caerulein-induced pancreatic tissue damage as indicated by the histologic damage score (Fig. 5a, b). Secondly, as shown in Fig. 5c, the pancreas-to-bodyweight ratio and the levels of serum amylase and lipase in NQO1^−/−^ mice also did not decrease (Fig. 5c). Third, we measured mRNA and protein levels of NOX isoforms, including NOX1, p22phox, p47phox, and p67phox. As expected, the levels of mRNA and protein of four NOX isoforms were increased in both caerulein and caerulein plus dunnione-coinjected NQO1^−/−^ mice (Fig. 6). Similarly, changes of ROS levels and cellular NADPH/NADP^+^ ratios were not affected by dunnione in the pancreas of NQO1^−/−^ mice (Fig. 7). In addition, the increase of IL-1β mRNA and protein levels were strongly induced in both caerulein and caerulein plus dunnione co-injected NQO1^−/−^ mice (Fig. 8). These results suggested that the protective effect of dunnione on acute pancreatitis is mediated by NQO1.

## Discussion

Acute pancreatitis is one of the most common gastrointestinal disorders characterized by pancreatic necrosis and inflammation. Despite its high morbidity and mortality rates, there is no specific and effective treatment for this disease^19^. In this study, we investigated whether ROS stress associated with NADPH oxidase (NOX) activity is affected by changes in the pancreatic NADPH/NADP^+^ ratio in a caerulein-induced acute pancreatitis mouse model. We also investigated whether the modulation of the cellular NADPH/NADP^+^ ratio by the enzymatic action of NQO1 is protective against acute pancreatitis through the regulation of NOX activity. We found that a decrease in the cellular NADPH/NADP^+^ ratio due to the enzymatic action of NQO1, which is activated by using dunnione as a substrate, downregulated the NOX activity, thereby reducing ROS production and pancreatic damage. Furthermore, as ROS production diminished, dunnione treatment markedly decreased pancreatic DNA damage. Of note, previous studies have demonstrated that dunnione undergoes an NQO1-dependent futile redox cycle^20,21^. This futile cycle leads to an imbalance in the redox cycle and induces intracellular ROS production in NQO1-overexpressing cancer cells. However, in vivo studies have demonstrated that dunnione has a protective effect against cisplatin-induced tissue damage through the downregulation of ROS^10,11^. In addition, pancreatic cells have lower NQO1 levels and are more insensitive to dunnione than cancer cells^22^. These findings might explain why the results of our study conflict with those of other studies with regards to the effect of dunnione on ROS production.

NOX has been suggested to be an important source of ROS in the early stages of acute pancreatitis^3,23^. NOX activity is regulated via two distinct mechanisms. The first pathway depends on the expression level of the NOX subunits. Various NOX subunits have been found to be present in pancreatic β cells^24,25^, pancreatic stellate cells^26,27^ and pancreatic acinar cells^4,28^. In particular, the NADPH oxidase subunits NOX1, p22phox, p47phox, and p67phox are expressed in pancreatic acinar cells, and their levels have been found to be increased in caerulein-stimulated pancreas through Jak2 activation^5^. Consistent with previous reports, our study also showed that the expression of NOX1, p22phox, p47phox, and p67phox NOX subunits increased by Jak2 activation in caerulein-induced acute pancreatitis, whereas this increase was attenuated by dunnione treatment. The second regulatory mechanism relies on NADPH concentration because NOX generates ROS by the reduction of molecular oxygen using NADPH as a common substrate. We found that increased pancreatic NOX activity in mice with caerulein-induced acute pancreatitis was dramatically suppressed by a decrease in NADPH level due to dunnione treatment. In addition, our in vivo studies with NQO1^−/−^ mice have further indicated that NQO1 activity is important for the inhibition of NOX activity by dunnione. Furthermore, we also found that the NOX1 inhibitor ML171 significantly reduced ROS production in caerulein-treated pancreatic acinar cells. These results suggest that the enzymatic action of activated NQO1 using dunnione as a substrate alleviates pancreatic oxidative stress induced by caerulein through modulating NOX activity by decreasing NADPH/NADP^+^ ratio and NOX expression.

There is increasing evidence that proinflammatory cytokines such as IL-1β, IL-6, and TNF-α play an important role in the inflammatory response associated with acute pancreatitis^29,30^. In vivo, studies have shown that infiltrating inflammatory cells play a crucial role in experimentally induced acute pancreatitis through the production of inflammatory mediators such as IL-1β and MCP-1^31,32^. In our experiment, dunnione treatment significantly reduced the infiltration of inflammatory cells and expression of inflammatory mediators in acute pancreatitis. This finding implies that dunnione treatment reduces the levels of inflammatory mediators by contributing to a reduction in inflammatory cell infiltration.

Meanwhile, previous in vitro studies have demonstrated the possible production of inflammatory cytokines in pancreatic acinar cells by caerulein stimulation without inflammatory cells^3,30^. In addition, NOX1 has been demonstrated to play a critical role in the induction of IL-6 in pancreatic acinar cells stimulated with caerulein^3^. We suggest that caerulein-induced IL-1β secretion can be induced by NOX1 activation, because the NOX1 inhibitor ML171 inhibited the secretion of caerulein-induced IL-1β in pancreatic acinar cells. Furthermore, ROS can act as an intracellular second messenger or chemoattractant to enhance the levels of cytokines, resulting in the aggravation of pancreatitis^33^. Thus, it is conceivable that the NOX1-induced production of inflammatory cytokines in the pancreas may increase pancreatic inflammation.

In conclusion, this is the first study showing that the activation of NQO1 by dunnione reduces pancreatic inflammation by reducing oxidative stress in acute pancreatitis. This reduction in oxidative stress is the result of reduced NOX enzyme activity, which in turn is caused by the NQO1 activation-associated reduction of the NADPH/NADP^+^ ratio. This study provides new insights into the mechanism of pancreatic protection associated with the regulation of NOX activity by NQO1 activation and identifies new potential targets and drugs for the treatment and prevention of acute pancreatitis.

## Supplementary information

Supplementary Information

## References

[1] Sendler M (2013). Tumour necrosis factor alpha secretion induces protease activation and acinar cell necrosis in acute experimental pancreatitis in mice. Gut.

[2] Willemer S, Elsasser HP, Adler G (1992). Hormone-induced pancreatitis. Eur. Surg. Res..

[3] Yu JH, Lim JW, Kim H, Kim KH (2005). NADPH oxidase mediates interleukin-6 expression in cerulein-stimulated pancreatic acinar cells. Int. J. Biochem. Cell. Biol..

[4] Kim H (2011). Inhibitory mechanism of lycopene on cytokine expression in experimental pancreatitis. Ann. N. Y. Acad. Sci..

[5] Yu JH, Lim JW, Kim KH, Morio T, Kim H (2005). NADPH oxidase and apoptosis in cerulein-stimulated pancreatic acinar AR42J cells. Free Radic. Biol. Med..

[6] Ross D (2000). NAD(P)H:quinone oxidoreductase 1 (NQO1): chemoprotection, bioactivation, gene regulation and genetic polymorphisms. Chem. Biol. Interact..

[7] Dolan ME (1998). Effects of 1,2-naphthoquinones on human tumor cell growth and lack of cross-resistance with other anticancer agents. Anticancer Drugs.

[8] Khambay BP, Batty D, Jewess PJ, Bateman GL, Hollomon DW (2003). Mode of action and pesticidal activity of the natural product dunnione and of some analogues. Pest Manag. Sci..

[9] Seo KS (2015). SIRT2 regulates tumour hypoxia response by promoting HIF-1alpha hydroxylation. Oncogene.

[10] Kim HJ (2016). Dunnione ameliorates cisplatin ototoxicity through modulation of NAD(+) metabolism. Hear. Res..

[11] Pandit A (2015). Dunnione ameliorates cisplatin-induced small intestinal damage by modulating NAD(+) metabolism. Biochem. Biophys. Res. Commun..

[12] Kim YH (2012). Prevention of salt-induced renal injury by activation of NAD(P)H:quinone oxidoreductase 1, associated with NADPH oxidase. Free Radic. Biol. Med..

[13] Leung PS, Chan YC (2009). Role of oxidative stress in pancreatic inflammation. Antioxid. Redox Signal..

[14] Lambeth JD (2004). NOX enzymes and the biology of reactive oxygen. Nat. Rev. Immunol..

[15] Fenyo IM, Florea IC, Raicu M, Manea A (2011). Tyrphostin AG490 reduces NAPDH oxidase activity and expression in the aorta of hypercholesterolemic apolipoprotein E-deficient mice. Vasc. Pharmacol..

[16] Yu JH, Kim KH, Kim H (2008). SOCS 3 and PPAR-gamma ligands inhibit the expression of IL-6 and TGF-beta1 by regulating JAK2/STAT3 signaling in pancreas. Int. J. Biochem. Cell. Biol..

[17] Hoque R, Malik AF, Gorelick F, Mehal WZ (2012). Sterile inflammatory response in acute pancreatitis. Pancreas.

[18] Hoque R (2011). TLR9 and the NLRP3 inflammasome link acinar cell death with inflammation in acute pancreatitis. Gastroenterology.

[19] Zerem E (2014). Treatment of severe acute pancreatitis and its complications. World J. Gastroenterol..

[20] Reinicke KE (2005). Development of beta-lapachone prodrugs for therapy against human cancer cells with elevated NAD(P)H:quinone oxidoreductase 1 levels. Clin. Cancer Res..

[21] Bian J (2015). Synthesis and evaluation of (+/-)-dunnione and its ortho-quinone analogues as substrates for NAD(P)H:quinone oxidoreductase 1 (NQO1). Bioorg. Med. Chem. Lett..

[22] Cullen JJ (2003). Dicumarol inhibition of NADPH:quinone oxidoreductase induces growth inhibition of pancreatic cancer via a superoxide-mediated mechanism. Cancer Res..

[23] Kim H (2008). Cerulein pancreatitis: oxidative stress, inflammation, and apoptosis. Gut Liver.

[24] Mohammed AM, Syeda K, Hadden T, Kowluru A (2013). Upregulation of phagocyte-like NADPH oxidase by cytokines in pancreatic beta-cells: attenuation of oxidative and nitrosative stress by 2-bromopalmitate. Biochem. Pharmacol..

[25] Rebelato E (2012). Expression of NADPH oxidase in human pancreatic islets. Life Sci..

[26] Hu R (2007). Ethanol augments PDGF-induced NADPH oxidase activity and proliferation in rat pancreatic stellate cells. Pancreatology.

[27] Masamune A, Watanabe T, Kikuta K, Satoh K, Shimosegawa T (2008). NADPH oxidase plays a crucial role in the activation of pancreatic stellate cells. Am. J. Physiol. Gastrointest. Liver Physiol..

[28] Yu JH, Kim KH, Kim DG, Kim H (2007). Diphenyleneiodonium suppresses apoptosis in cerulein-stimulated pancreatic acinar cells. Int. J. Biochem. Cell. Biol..

[29] Norman J (1998). The role of cytokines in the pathogenesis of acute pancreatitis. Am. J. Surg..

[30] Yu JH, Lim JW, Namkung W, Kim H, Kim KH (2002). Suppression of cerulein-induced cytokine expression by antioxidants in pancreatic acinar cells. Lab. Invest..

[31] Bhatia M (2000). Inflammatory mediators in acute pancreatitis. J. Pathol..

[32] Mayer J, Rau B, Gansauge F, Beger HG (2000). Inflammatory mediators in human acute pancreatitis: clinical and pathophysiological implications. Gut.

[33] Fujimori N (2011). Vasoactive intestinal peptide reduces oxidative stress in pancreatic acinar cells through the inhibition of NADPH oxidase. Peptides.

