# Supplementary material for: Pharmacological stimulation of NQO1 decreases NADPH levels and ameliorates acute pancreatitis in mice
Source: Cell Death Dis. 2018 Dec 18;10(1):5. doi: 10.1038/s41419-018-1252-z (PMC6315021; doi:10.1038/s41419-018-1252-z)
Supplement: Supplementary file 1 — Supplementary Information [file 41419_2018_1252_MOESM1_ESM.pdf]

Pharmacological stimulation of NQO1 decreases NADPH levels and ameliorates acute pancreatitis in mice

AiHua Shen[1,*], Hyung-Jin Kim[1,*], Gi-Su Oh[1], Su-Bin Lee[1], SeungHoon Lee[1], Arpana Pandit[1], Dipendra Khadka[1], Subham Sharma[1], Seon Young Kim[1], Seong-Kyu Choe[1], Sei-Hoon Yang[2], Eun-Young Cho[2], Hyuk Sim[2], Raekil Park[3], Tae Hwan Kwak[1], Hong-Seob So[1, #]

[1]Center for Metabolic Function Regulation & Department of Microbiology, [2]Internal Medicine, Wonkwang University School of Medicine, Iksan, Jeonbuk 54538, Republic of Korea. [3]Department of Biomedical Science & Engineering, Institute of Integrated Technology, Gwangju Institute of Science and Technology, Gwangju 61005, Republic of Korea.

Running title: Impact of NADPH levels in acute pancreatitis

[#]Correspondence: Hong-Seob So, Department of Microbiology, Wonkwang University School of Medicine, 460 Iksan-Daero, Iksan, Jeonbuk 54538, Republic of Korea.

E-mail: jeanso@wku.ac.kr; Phone: 82-63-850-6950; Fax: 82-63-855-6777.

*These authors contributed equally to this work.

Supplementary figure legends

Fig. S1. **NQO1 activity was increased by dunnione.** Freshly prepared pancreatic acinar cells were treated with dicumarol or dunnione. NQO1 activity was analyzed by measuring the conversion rate of NADH to NAD^+ using 2,6-dichlorophenolindophenol. Each value represents the mean ± SD (n=5). **P< 0.01.

Fig. S2. **Protective effect of dunnione on ROS generation and DNA damage is mediated by NQO1.** Freshly prepared pancreatic acinar cells were treated with dicumarol or dunnione and cultured in the presence of caerulein for 12 h. (A) NOX activity was measured by cytochrome c reduction assay. (B) ROS levels were measured using a fluorometer and normalized to protein content. (C) Cell viability was determined by crystal violet staining assay. (D) Representative images and quantification of damaged DNA in the comet assay. Each value represents the mean ± SD (n=5). *P< 0.05, **P< 0.01. Scale bar: 20 μm.

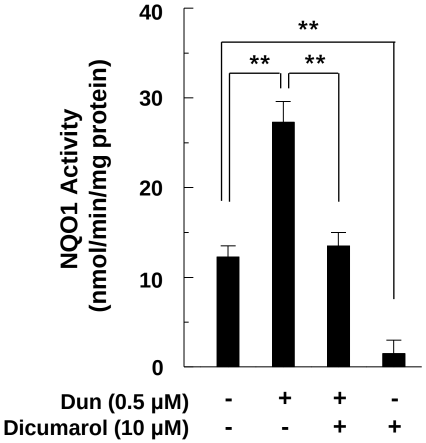

Supplementary Fig. S1

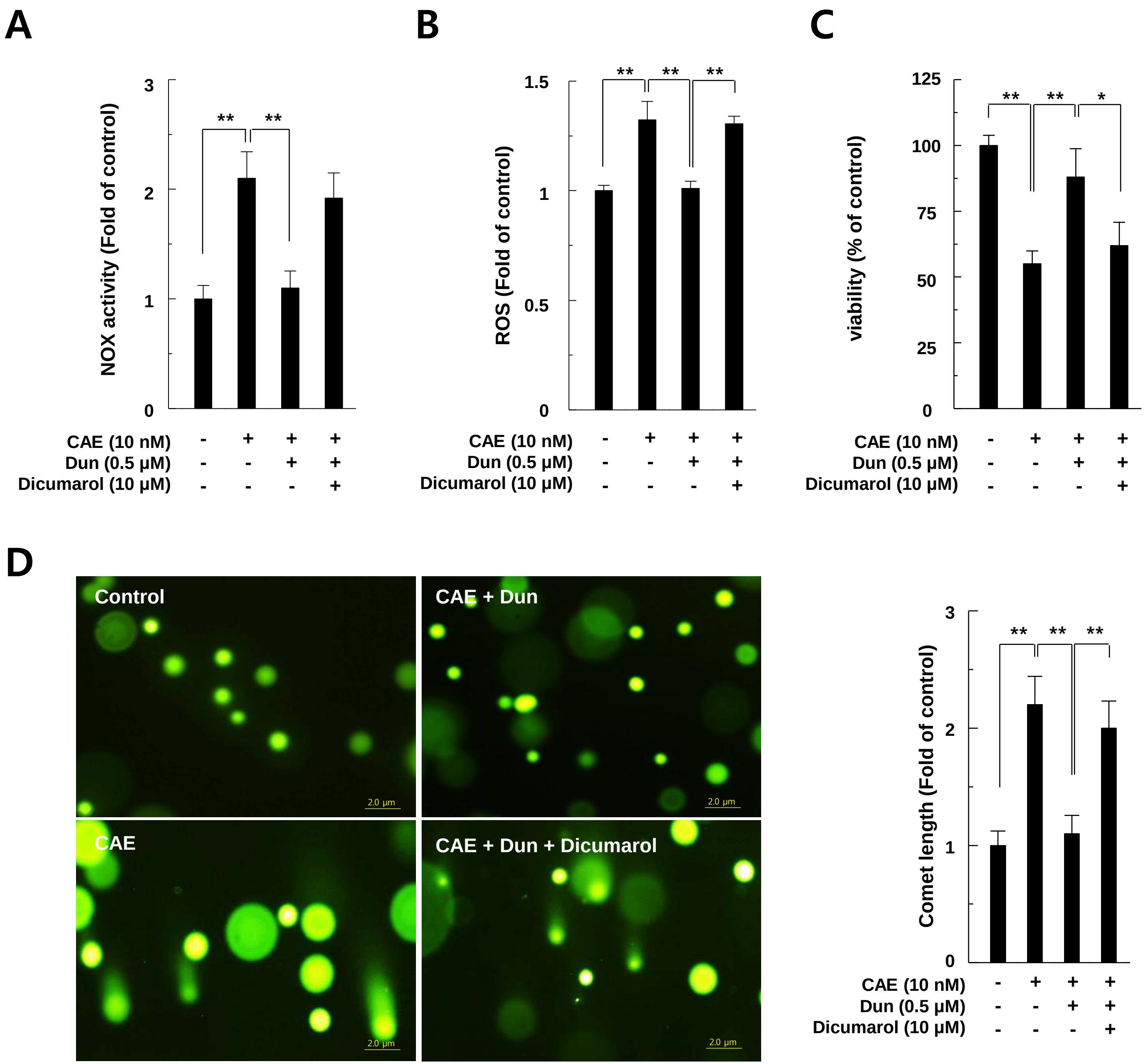

Supplementary Fig. S2
